# Application of L-moment method for regional frequency analysis of meteorological drought across the Loess Plateau, China

**DOI:** 10.1371/journal.pone.0273975

**Published:** 2022-09-01

**Authors:** Ming Li, Meilin Liu, Fuqiang Cao, Guiwen Wang, Xurong Chai, Lianzhi Zhang

**Affiliations:** 1 College of Geographical Science, Shanxi Normal University, Taiyuan, Shanxi, China; 2 Library of Shanxi Normal University, Shanxi Normal University, Taiyuan, Shanxi, China; Institute of Earth and Environment, Chinese Academy of Sciences, CHINA

## Abstract

Water shortages have always been the primary bottleneck for the healthy and sustainable development of the ecological environment on the Loess Plateau (LP). Proper water resource management requires knowledge of the spatiotemporal characteristics of precipitation frequency. This paper employed the gridded precipitation dataset obtained from the China Meteorological Data Service Centre to present a spatially explicit characterization of precipitation frequencies in tandem with their return periods on the LP based on the L-moment method. The 60% and 80% of the mean annual precipitation from 1981 to 2010 were synonymous with severe and moderate droughts, respectively. Droughts occurred more frequently in the northwest than in the southeast of the LP. Moreover, the frequencies of moderate drought showed a slight difference throughout the area, while those of severe droughts demonstrated considerable differences between the northwestern arid zone and the southeastern semi-humid zone. The maps associated with various return periods of precipitation deficits can be used to produce drought risk maps together with drought vulnerability maps. These findings could also provide useful information for drought management, water resource management and the development of food security policies.

## Introduction

Drought is a common and recurring phenomenon that occurs almost everywhere. It usually refers to the deficit of precipitation compared to what is considered "normal" for a specific duration [1]. Drought is different from aridity, a permanent climatic feature in arid areas [2]. China is a drought-prone country that has suffered from many severe droughts during its history. For instance, the noticeable drought, which occurred in 1637–1643, indirectly led to the extinction of the Ming Dynasty. Another severe drought, accompanied by severe locust plagues, killed more than 3 million lives from famine and diseases during the late 1920s to early 1930s [3]. Since the founding of the People’s Republic of China, China has also experienced severe droughts in the late 1960s, late 1970s, early 1980s, and late 1990s [4].

The Loess Plateau (LP) is located in northwestern China, where the spatiotemporal distributions of precipitation are extremely uneven due to the sizeable climatic zone from the semi-humid zone to the semi-arid and arid zones, as well as the complex topography. Water shortages have historically been the primary bottleneck for the healthy and sustainable development of the ecological environment on the LP. In the context of global warming, numerous scholars [5–7] have pointed out a significant upward trend in temperature and a non-significant downward trend in precipitation. The changes in temperature and precipitation cause the drought frequency to increase across the LP, resulting in substantial agricultural and socio-economic losses [8].

Precipitation is a crucial factor in drought occurrence because rainfall variations determine the intensity and influence range of droughts [9]. Therefore, it is a prerequisite to learn about the amount and distribution of precipitation in a region for effective water resource management. For example, in the agricultural sector, detailed knowledge of precipitation patterns is necessary to identify the most appropriate crop varieties for the region and to manage climate-related uncertainties effectively [10]. Understanding the distribution of precipitation can also reveal the areas most prone to increased extreme events, whether floods or droughts. However, the drought frequency analysis in many previous studies [11–14] was commonly based on at-site estimates, which do not adequately describe the drought potential because of its regional properties [15]. A number of researchers [1,10,16,17] have emphasized the importance of regional drought frequency, which brings the stations having similar drought characteristics to form a homogeneous region. Regional frequency analysis (RFA) can get over the limitations of statistical estimation, such as the absence of lengthy or too short records, and result in more accurate quantile estimates compared to at-site analysis. Therefore, RFA has attracted increasing attention from researchers [18–20].

To carry out RFA, the considered region should be homogeneous; otherwise, it should be subdivided into some homogeneous regions. Some scholars [5,8,21] have analyzed the temporal evolution of droughts on the LP based on regional classification using the orthogonal function decomposition method and hierarchical clustering method. However, none of them were concerned with the drought frequency or return period by applying RFA in spatially well-defined homogeneous regions. Moreover, it should be noted that homogeneity tests are necessary when using RFA. According to the previous literature [19,20,22], the L-moment methods proposed by Hosking & Wallis [16] are the most well-known. Subsequently, many researchers [19,20,22,23] used the L-moments to carry out the RFA on hydrological phenomena, such as rainfall, drought, streamflow, and so forth.

Previous studies on precipitation on the LP have mainly concentrated on its spatial and temporal variability [24–28]. Additionally, several authors [29–31] have applied the regional frequency analysis based on L-moment method (RFA-LM) to quantitatively reveal the characteristics of extreme precipitation on the LP. However, little work has been done based on the RFA-LM to map drought return periods spatially for different drought levels in the study area. The spatial maps obtained can be an effective tool for drought risk management program planning and implementation. This aspect has been taken into consideration by some studies in drought analysis, such as mapping the return period of drought spells in northeastern Spain [32] and mapping the annual maximum dry spell length at different return periods in Iran [33]. Therefore, the primary aims of this study are to (1) analyze meteorological drought at a regional scale based on RFA-LM; (2) assess both severe and moderate droughts corresponding to 60% and 80% of the mean annual precipitation (MAP), respectively; and (3) map the return periods of meteorological drought for revealing the most drought-prone regions in the LP.

## Materials and methods

### Study area

**Fig 1:**
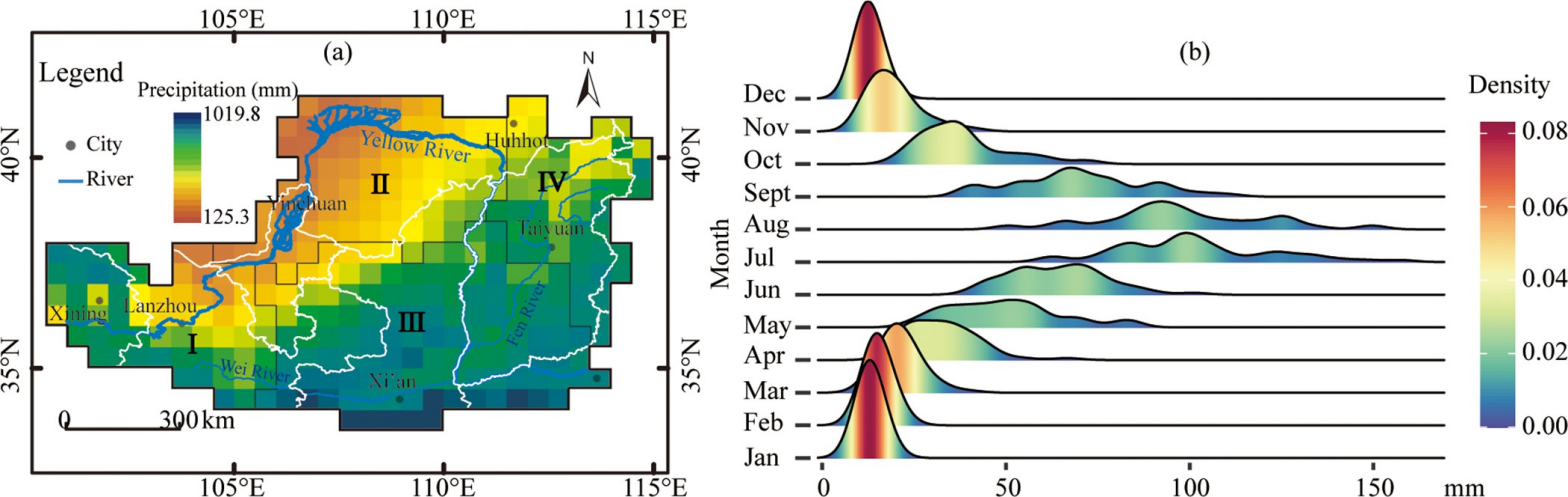
Mean annual precipitation (a) and kernel density estimates of monthly precipitation (b) for the Loess Plateau in China during 1961–2017. In Fig 1b, the x-axis relates to the numerical range of precipitation, the y-axis corresponds to different months, and the color is mapped to the kernel density estimate of each monthly rainfall.

The LP is situated between 33°43′-41°16′N and 100°54′-114°33′E, with an area of approximately 6.24×10^5^ km^2^. It starts from the Taihang Mountains in the east, reaches the Riyue Mountains in the west, and borders the Yinshan Mountains in the north and the Qinling Mountains in the south with an elevation ranging from 75 m to 5149 m a.s.l. [8]. The northwestern part of the region is dominated by flat sandy areas, and the middle and southeastern parts are characterized by rolling gullies and hills [24]. The region has typical continental monsoon climate characteristics (hot and rainy summer, dry and cold winter). The average annual temperature increases gradually from 3.6°C in the northwest to 14.3°C in the southeast [8], and the MAP increases from 125 mm to 1020 mm in a similar direction (Fig 1A). The seasonal distribution of rainfall is uneven (Fig 1B), accounting for 60%-80% from July to September. In addition, the annual and seasonal precipitation variability is large due to the monsoon’s influence. The relative variability of annual precipitation in the region is 20%-30% on average, and that of seasonal precipitation is more than 50%-90%. Rainfall variability has been considered a considerable cause of historic food security and reduced productivity [10]. Generally, the vegetation zones change with the precipitation from southeast to northwest in the order of forest, forest-steppe, typical-steppe, desert-steppe, and steppe-desert zones [8].

### Data collection

In this study, the 0.5°×0.5° gridded monthly rainfall dataset of the LP for the period 1961–2017 was obtained from the China Meteorological Data Service Centre (http://www.data.cma.cn). The dataset was interpolated using the thin-plate smooth spline method of ANUSPLIN software. All the gridded points underwent strict quality inspection, quality control, data verification, data correction, and supplementary recording. As a result, the root-mean-square error between the grid values and the at-site observation values is 0.49 mm on average, and the correlation coefficient between them is 0.93 (arriving at the significance of α = 0.01) on average [34]. Therefore, the gridded dataset has excellent accuracy. For subsequent analysis, we merged the monthly precipitation data into annual precipitation data. In addition, the Loess Plateau boundary shapefile [35] was downloaded for free from the Global Change Research Data Publishing & Repository (http://geodoi.ac.cn/WebCn/doi.aspx?Id=199). All data processing and calculations were performed using R statistical software (R Development Core Team 2008), and the visualizations were presented using ArcGIS 10.4 software (Environmental Systems Research Institute, Redlands, California, United States).

### Methodology

The RFA-LM procedure proposed by Hosking & Wallis [16] was employed in this study. L-moments, derived from probability-weighted moments, are the pillars of RFA. Given an ordered sample of size *n*, such that $\left[X_{1:n}\leq X_{2:n}\leq X_{3:n}\cdots \leq X_{n:n}\right]$ the L-moments of a probability distribution are expressed as

$$\lambda_{r}=r^{-1}\sum_{j=0}^{r-1}{\left(-1\right)}^{j}(\begin{array}{c} r-1\\ j \end{array})E(X_{r-j:r})$$ (1)

The expectation of an order statistic is defined by

$$E(X_{r:n})=\frac{n!}{(r-1)!(n-r)!}\int_{0}^{1}x(u)u^{r-1}{\left(1-u\right)}^{n-r}du$$ (2)

Specifically, for the first four L-moments:

$$\lambda_{1}{=E(X}_{1:1})$$ (3)

$$\lambda_{2}=\frac{1}{2}{E(X}_{2:2}-X_{1:2})$$ (4)

$$\lambda_{3}=\frac{1}{3}{E(X}_{3:3}-2X_{2:3}{+X}_{1:3})$$ (5)

$$\lambda_{4}=\frac{1}{4}{E(X}_{4:4}-3X_{3:4}+3X_{2:4}{‐X}_{1:4})$$ (6)

Finally, the L-moment ratios are calculated as:

$$L‐\mathrm{mean}=\tau_{1}=\lambda_{1}$$ (7)

$$L‐\mathrm{cv}=\tau_{2}=\frac{\lambda_{2}}{\lambda_{1}}$$ (8)

$$L‐\mathrm{skewness}=\tau_{3}=\frac{\lambda_{3}}{\lambda_{2}}$$ (9)

$$L‐\mathrm{kurtosis}=\tau_{4}=\frac{\lambda_{4}}{\lambda_{2}}$$ (10)

where L-mean is the same as the conventional statistical mean, and L-cv measures the dispersion of variables, i.e., the expected difference between two random samples. L-skewness and L-kurtosis are measures related to the shape of the samples distribution. The L-skewness quantifies the asymmetry of the samples distribution, and the L-kurtosis measures whether the samples are peaked or flat relative to a normal distribution [10,16].

Five steps are generally needed to perform the RFA-LM procedure. The procedures are summarized below.

#### Step 1: Data screening and quality assessment

The precipitation dataset used in this study was developed by Zhao et al. [34], who constructed a monthly gridded precipitation dataset with a resolution of 0.5°×0.5° based on 2472 observed stations across China. They evaluated the data accuracy based on generalized cross-validation, root-mean-square error, mean bias error, relative bias error, and so on. The results illustrated that the gridded datasets had excellent accuracy and an ideal interpolation effect. Therefore, numerous scholars [5,36,37] have used this dataset to study climate-related research. In addition, we checked the stationarity of annual precipitation across the LP for the period 1961–2017 using the Kwiatkowski-Phillips-Schmidt-Shin (KPSS) test [38].

#### Step 2: Recognition of homogeneous sub-regions

It is the prerequisite and basis for the RFA-LM to identify homogeneous sub-regions in which the precipitation data can be depicted using the same probability distribution. The homogeneity of each sub-region was confirmed by employing the H_1_ heterogeneity measure (for more information about heterogeneity measures, see reference [16]). The H_2_ and H_3_ heterogeneity measures were not used, because their values are rarely larger than 2 even for grossly heterogeneous regions and hence have a weak ability to distinguish homogeneous regions and heterogeneous regions [16,39,40]. A region is considered to be ’acceptably homogeneous’ when H_1_<1, ’possibly homogeneous’ when 1≤H_1_<2, and ’heterogeneous’ when H_1_≥2. In this study, we adopted the regionalization results concluded by Li et al. [5]. They employed the grid characteristics, namely, latitude, longitude, altitude, precipitation concentration degree (PCD), and precipitation concentration period (PCP) as the input data for the hierarchical clustering analysis. Latitude, longitude, and elevation were used to ensure the continuity of the identified homogeneous sub-regions. The PCD takes values from 0 to 1. Values close to 0 indicate a wide variation in precipitation in one year, while values near 1 indicate a small deviation in precipitation within a year. The PCP is the arithmetic mean for dates, which denotes the average time of most rainfall occurrences [1]. The PCD and PCP measures have been employed successfully by Núñez et al. [1] for identifying homogeneous sub-regions.

#### Step 3: Choice of the regional probability distribution

L-moment ratio diagrams (L-kurtosis vs. L-skewness) were employed to perform a goodness-of-fit assessment and identify the best-fit regional probability distribution for the homogeneous sub-regions. A distribution was considered optimal if the distance between the fitted L-moments and the sample L-moments was the shortest. Vogel & Wilson [41] also described several advantages of the L-moment ratio diagram in choosing an appropriate probability distribution. In this study, five commonly applied 3-parameter distributions, namely, the generalized logistic (GLO), generalized extreme value (GEV), generalized normal (GNO), generalized Pareto (GPA), and Pearson type III (PE3), were examined.

#### Step 4: Parameters and quantiles estimation

Once the regional probability distribution for each homogeneous sub-region was identified, the distribution parameters were estimated using the L-moments described by Hosking & Wallis [16]. The L-moment ratios for each grid can be computed based on Eqs (8)—(10). Then, the regional growth curve can be generated according to the distribution parameters. The quantile estimates $\hat{Q}(F)$ with non-exceedance probability *F* at a grid in a sub-region are computed by Eq (11).

$${\hat{Q}}_{i}(F)={\hat{\mu }}_{i}\hat{q}(F)$$ (11)

where ${\hat{\mu }}_{i}$ is the MAP for grid *i* and $\hat{q}(F)$ is the regional growth curve.

#### Step 5: Spatial mapping of L-moment ratios and drought characteristics

Spatial mapping of annual precipitation for various return periods is helpful in evaluating the drought risk throughout the study area. The return period (*T*) can be computed as:

$$T=\frac{1}{1-F}$$ (12)

where *F* is the non-exceedance probability mentioned in step 4. The values *F* = 0.8, *F* = 0.9, *F* = 0.98, and *F* = 0.99 correspond to return periods of 5-, 10-, 50-, and 100-year, respectively. The estimates for annual precipitation in a specific return period for each grid can be obtained by multiplying the regional growth curve by the observed MAP (Eq (11)). The return period maps for 80% and 60% of the normal (20% and 40% deficits) annual precipitation were also presented in the study, respectively. The ’normal’ stands for the MAP from 1981–2010. This result is consistent with the concept of defining drought thresholds as a percentage of the most recent 30-year climatic normal for MAP [1]. In this study, 80%-of-normal and 60%-of-normal are synonymous with moderate and severe droughts, respectively.

## Results

### Data screening and assumptions checking

Table 1 presents the summary statistics for annual precipitation from the 311 efficient grids on the LP. The results showed that the average MAP is 456.9 mm, with a minimum of 125.3 mm and a maximum of 1019.8 mm. The stationarity test results indicated that 96.5% of the grids passed the significance level (α = 0.05). Therefore, the time series of annual precipitation data could be considered to be stationary.

**Table 1:** Summary statistics for mean annual precipitation (MAP) of the 311 grids.

Variable	Value
Average MAP (mm)	456.9
Minimum MAP (mm)	125.3
Maximum MAP (mm)	1019.8
Range of the MAP (mm)	894.5
Standard deviation of the MAP (mm)	156.2
Total efficient grids	311

### Regionalization

**Fig 2:**
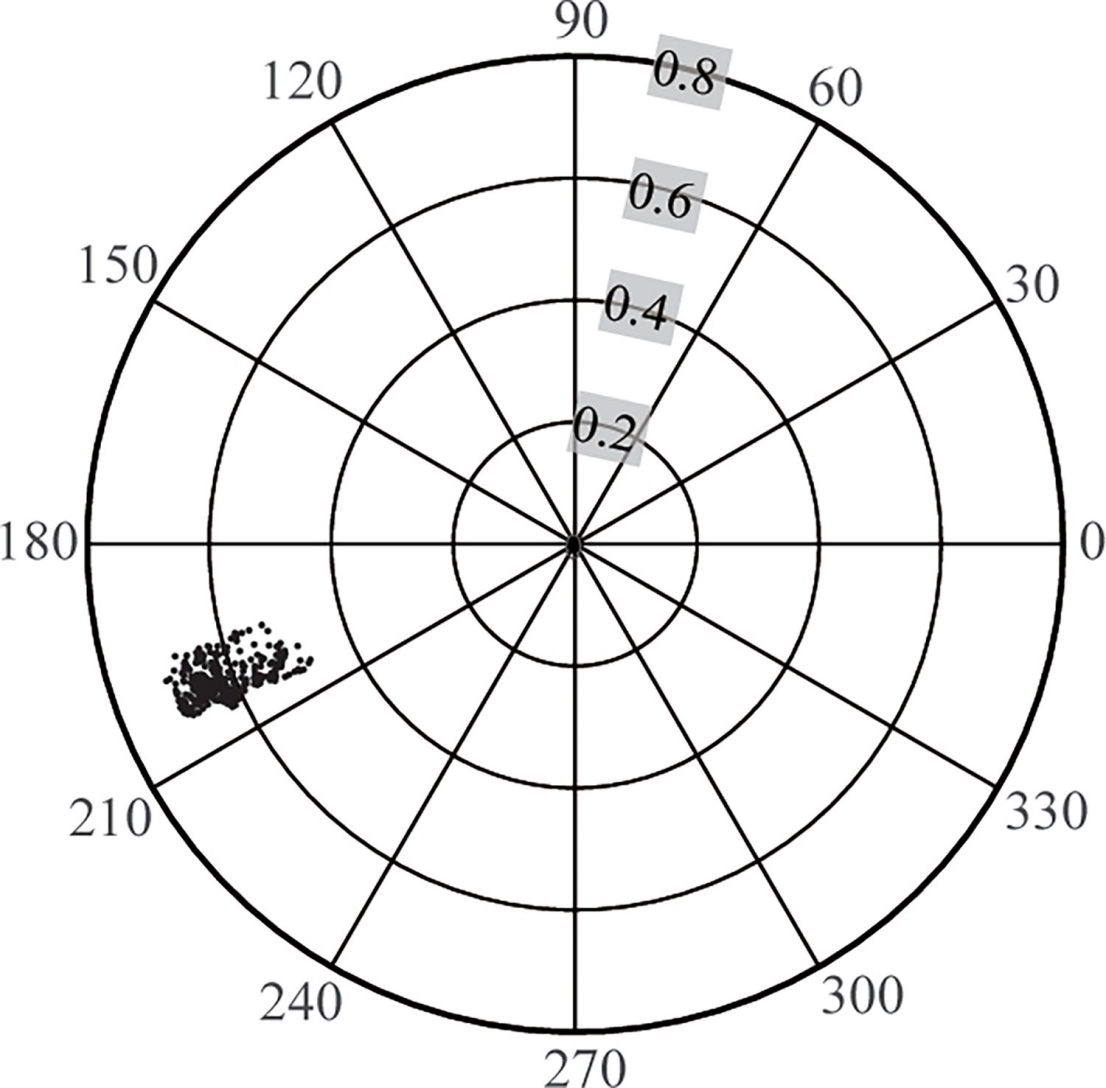
Location of the precipitation concentration degree and precipitation concentration period on the Loess Plateau. The angles mean: 0°-January 1^st^, 90°-April 1^st^, 180°-July 1^st^, and 270°-October 1^st^.

In this study, the PCD values vary from 0.46 to 0.70, with an average of 0.61. This means that the rainfall on the LP is concentrated in a few months. The PCD ranged from Day 198 to 210 (Fig 2), with an average of Day 204. That is, a high concentration of rainfall is mainly in mid-late July on the LP. As a result, four spatially well-defined homogenous regions were identified (Fig 1A), and the summary statistics of each sub-region are listed in Table 2. The four sub-regions are all acceptable homogeneous regions because the H_1_ heterogeneity measures derived from L-moments are less than 1 [5].

**Table 2:** Characteristics of the four homogeneous sub-regions.

Sub-region	Region Name	MAP (mm)	Number of Grids	L-cv	L-skewness	L-kurtosis	H_1_
Ⅰ	Longzhong plateau cold-arid area	445.0	62	0.099	0.065	0.115	-0.06
Ⅱ	Ordos Plateau semi-arid area	280.1	78	0.154	0.055	0.111	-0.13
Ⅲ	Fenwei Plain and Shaanxi-Shanxi hilly semi-humid area	567.7	119	0.113	0.079	0.137	-0.12
Ⅳ	Northern Shanxi hilly semi-humid area	482.8	52	0.117	0.053	0.152	-0.24

### Regional probability distribution selection

**Fig 3:**
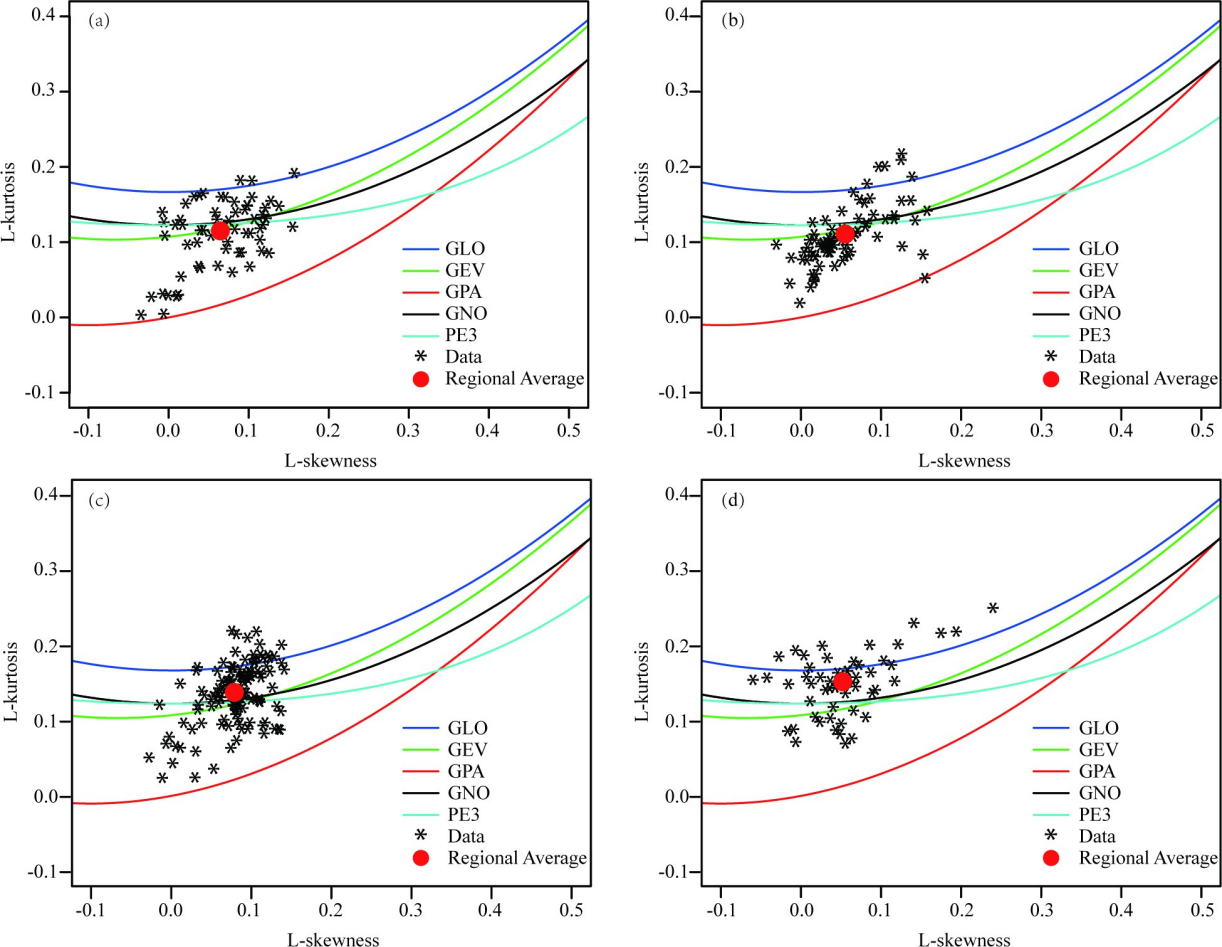
L-moment ratio diagrams of L-skewness vs. L-kurtosis for the homogeneous sub-regions (a) Ⅰ, (b) Ⅱ, (c) Ⅲ, and (d) Ⅳ. The red squares indicate the regional average L-moments.

Fig 3 presents the L-moment ratio diagrams with the regional average L-moment ratio for the four homogeneous sub-regions across the LP. The best-fit models are the GEV distribution for regions Ⅰ and Ⅱ, the GNO distribution for region Ⅲ, and the GLO distribution for region Ⅳ. The results do not suggest any unique parent frequency distribution across the entire domain, mainly because of the complexity of rainfall mechanisms caused by the combined effects of elevation, topography, large atmospheric systems, etc. [42].

### Parameters and quantiles estimation

Table 3 displays the location, scale, and shape parameters (ξ, α, κ) of the selected distributions computed using average regional L-moment ratios. Once the distribution parameters are available, the regional quantiles of annual precipitation and their associated frequencies for the four homogeneous sub-regions can now be estimated [33]. Table 4 shows the variability in quantile estimates reduced from arid and semi-arid regions in the northwest to semi-humid zones in the southeast. It is associated with a decreasing trend from northwest to southeast of L-cv, as presented in Table 2. Therefore, more frequent extremes can be expected in the drier areas than in the wetter ones of the study area. A similar result was reported from a study by Wallis et al. [43]. This result also agrees with the fact that there is high inter-annual rainfall variability within arid and semi-arid regions [1]. For instance, a 0.8 quantile of the regional growth curve (equivalent to 80%-of-normal) had a probability of non-exceedance of 0.16 in sub-region Ⅲ, equivalent to a return period of approximately 6 years (100/16). In contrast, the same quantile in sub-region Ⅱ had a probability of non-exceedance of 0.25, equivalent to a return period of approximately 4 years. A 0.6 quantile of the regional growth curve (equivalent to 60%-of-normal) had a probability of non-exceedance of 0.01 in sub-region Ⅲ, equivalent to a return period of approximately 100 years. However, the same quantile in sub-region Ⅱ had a probability of non-exceedance of 0.06, equivalent to a return period of about 17 years. In fact, the differences are even more considerable in the lower and upper tail of the regional growth curves. These results are likely to be related to the rainfall variability of each sub-region.

**Table 3:** Regional parameter estimates for the selected distribution.

Sub-region	Distribution	Parameters		
		ξ	α	κ
Ⅰ	GEV	0.930	0.163	0.170
Ⅱ		0.892	0.257	0.187
Ⅲ	GNO	0.984	0.197	-0.162
Ⅳ	GLO	0.990	0.116	-0.053

**Table 4:** Regional growth curve for the four homogeneous sub-regions.

Non-exceedance probability, *F*	Sub-region, $\hat{q}(F)$				Non-exceedance probability, *F*	Sub-region, $\hat{q}(F)$			
	Ⅰ	Ⅱ	Ⅲ	Ⅳ		Ⅰ	Ⅱ	Ⅲ	Ⅳ
0.001	0.556	0.295	0.504	0.316 0.374	0.550 0.600	1.010 1.033	1.018 1.055	1.009 1.035	1.013 1.038
0.002	0.579	0.333	0.530						
0.005	0.615	0.390	0.568	0.453	0.650	1.058	1.093	1.062	1.063
0.010	0.645	0.439	0.602	0.515	0.700	1.084	1.133	1.092	1.091
0.020	0.679	0.494	0.639	0.581	0.750	1.113	1.178	1.125	1.121
0.050	0.732	0.580	0.699	0.673	0.800	1.146	1.228	1.162	1.157
0.100	0.783	0.661	0.756	0.749	0.850	1.185	1.288	1.207	1.201
0.150	0.819	0.718	0.796	0.797	0.900	1.235	1.364	1.265	1.261
0.200	0.849	0.765	0.829	0.834	0.950	1.310	1.478	1.356	1.360
0.250	0.875	0.806	0.858	0.866	0.960	1.333	1.511	1.383	1.392
0.300	0.899	0.844	0.885	0.894	0.980	1.395	1.604	1.465	1.492
0.350	0.922	0.880	0.910	0.919	0.990	1.451	1.685	1.541	1.594
0.400	0.944	0.915	0.935	0.943	0.995	1.500	1.756	1.614	1.700
0.450	0.966	0.949	0.959	0.967	0.998	1.556	1.837	1.707	1.844
0.500	0.988	0.983	0.984	0.990	0.999	1.593	1.889	1.775	1.958

### Spatial mapping

**Fig 4:**
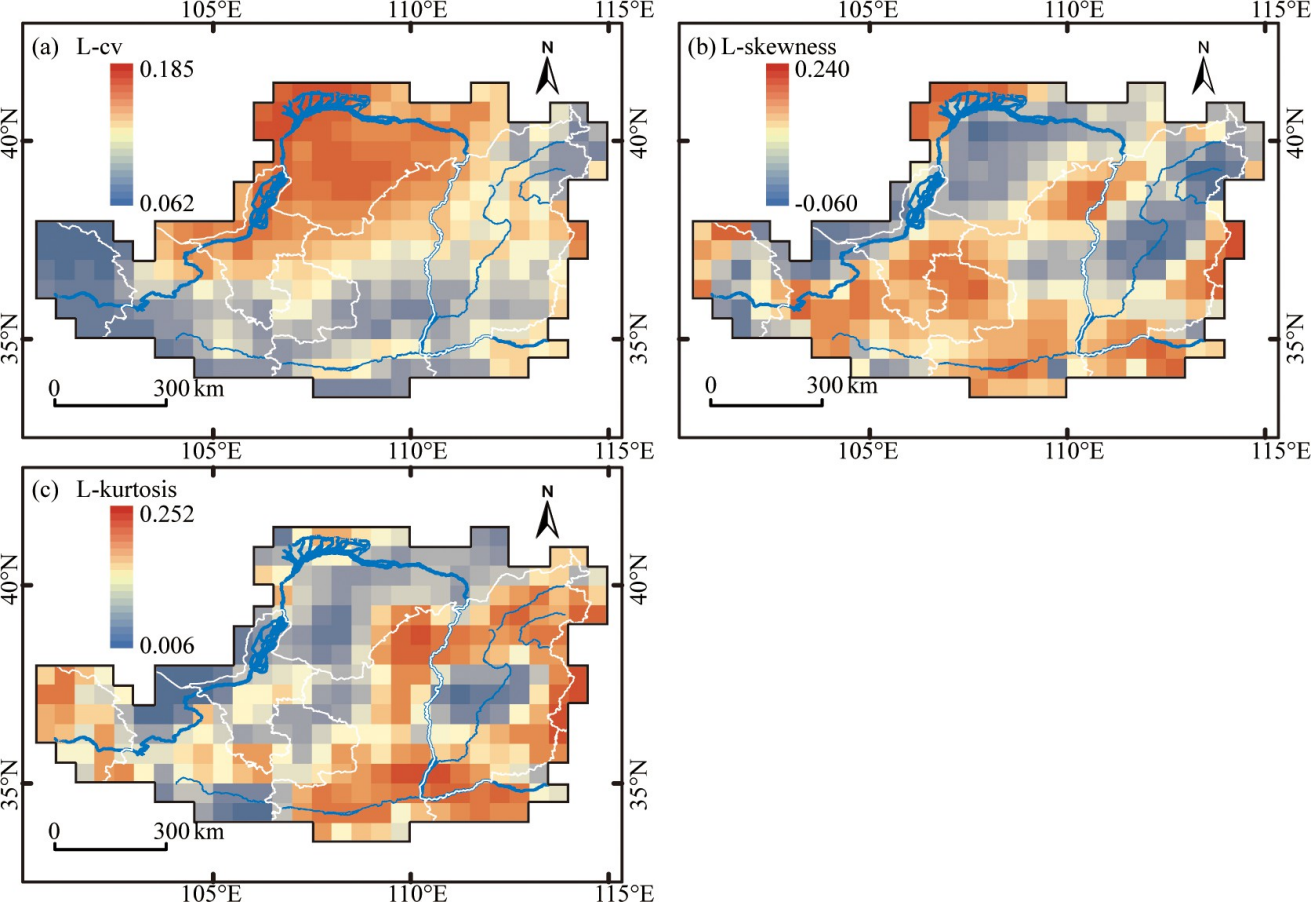
Spatial patterns of annual precipitation L-moments over the Loess Plateau (a) L-cv, (b) L-skewness, and (c) L-kurtosis. L-cv measures the dispersion of precipitation, whereas L-skewness and L-kurtosis reflect the shape of the sample distribution.

Fig 4 presents the spatial distribution of the L-moment statistics over the LP. The L-cv values were generally higher in semi-arid and arid regions, and they offered an increasing trend from southeast to northwest (Fig 4A). The probable reason is that the moment ratio increases with a lower MAP and small dispersions considerably impact sample variance [10]. Spatial variation in the L-skewness demonstrates negative values in rainless regions, especially in the northwestern Ordos Plateau and northern Shanxi Province, while positive values are demonstrated in relatively rainy zones and cold-arid areas, i.e., the southwestern LP (Fig 4B). The L-skewness roughly shows an increase along the north-south axis. In addition, high values of L-kurtosis are mainly in the southeast, where the most precipitation is observed (Fig 1A). In contrast, low L-kurtosis values are found in regions with less annual precipitation (Fig 4C). Approximately, a decreasing trend of L-kurtosis was detected in the southeast-northwest direction. Similar results can also be seen in Table 2.

**Fig 5:**
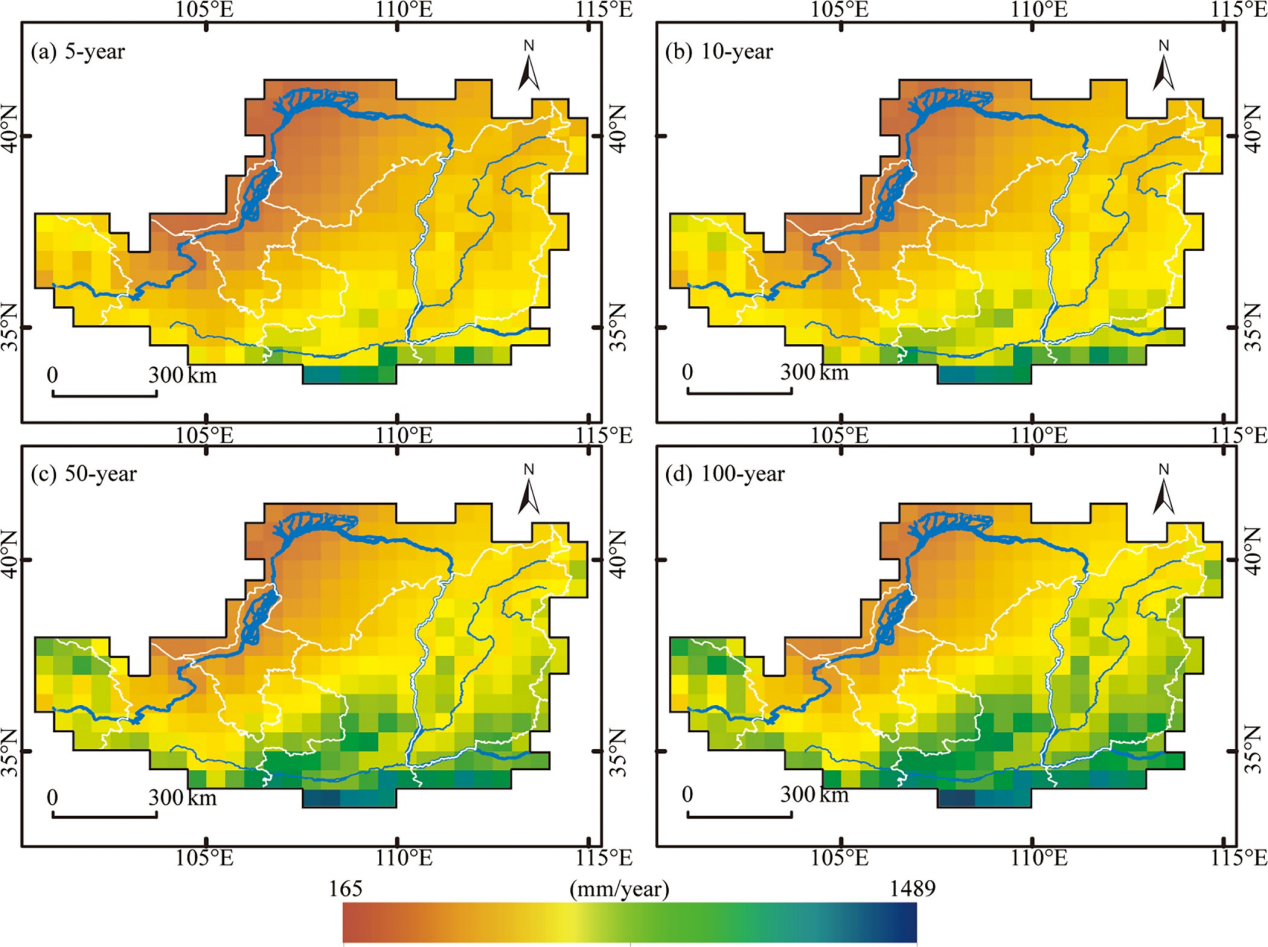
Expected annual precipitation for various return periods, (a) 5-year, (b) 10-year, (c) 50-year and (d) 100-year, from 1961 to 2017.

Fig 5 shows the spatial pattern of the 5-, 10-, 50-, and 100-year return values of annual precipitation. As expected, the amount of annual precipitation increases with the increasing return period. It can also be seen from this figure that the precipitation maps associated with different return periods have a similar spatial pattern. This result is also consistent with the spatial distribution of MAP (as shown in Fig 1A). According to the results presented in Figs 1A and 5, the rainy areas are detected mainly in the southeastern LP, while the less rainy areas are found mainly in the northwestern LP (particularly in the Ordos Plateau). A possible explanation for this might be that the LP is at the edge of the East Asian summer monsoon. When the warm, humid air mass travels from south to north, it is hindered by the Qinling Mountains and the LP, and the water vapor mass fraction decreases, thus forming a pattern of more precipitation in the southeast and less precipitation in the northwest.

**Fig 6:**
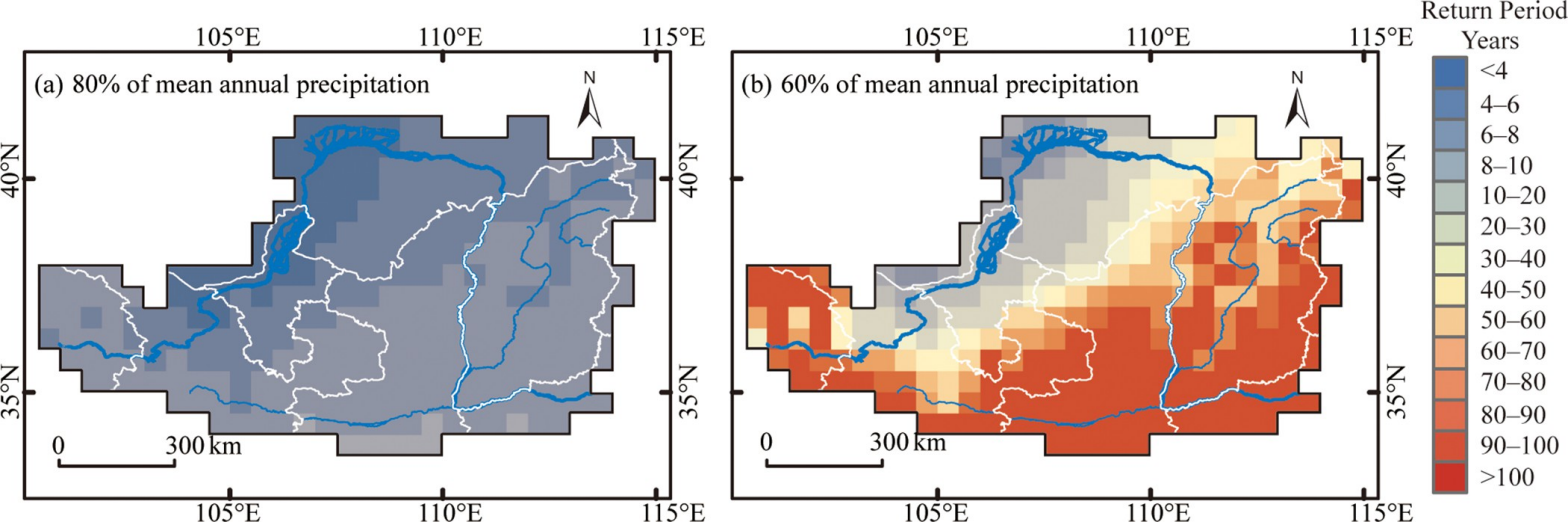
Spatial patterns of the return periods for (a) 80%-of-normal and (b) 60%-of-normal annual precipitation. The ’normal’ stands for the mean annual precipitation from 1981–2010, and the 80%-of-normal and 60%-of-normal are synonymous with moderate and severe droughts in this study.

Fig 6A and 6B show the drought return period maps for 80% and 60% of the normal (20% and 40% deficits) annual precipitation, respectively. The results indicate that the return periods of moderate drought range from 2 years to 8 years, with a minimum of 2 years around the northwest edge and approximately 8 years at the southeast edge. The reason that the return period is not much different is due to the slight differences between the quantiles to the regional growth curves around the central values of the distribution (seen from Table 4). In contrast, the return periods of severe drought are between 4 and 10 years on average at the northwest edge and more than 100 years at the southeast border. These results imply that higher aridity corresponds to more extreme annual drought events [1]. The maps also show an increasing drought frequency from southeast to northwest, associated with decreasing annual precipitation in that direction. As known, the northwestern part of the LP is predominantly associated with livestock raising except for the Hetao region, while rain-fed agriculture is dominant in the southeastern part of the study area, mainly including the Fenwei Plain. In addition, droughts of 60%-of-normal do not cause the same impact on the southeast and northwest of the LP (Fig 6B). These results suggest that different drought thresholds need to be set for different parts of the study area [1].

## Discussion

Numerous authors [1,16,23] have used L-moment ratio diagrams for their selection of regional probability distributions. L-moment ratio diagrams have been suggested as a powerful tool for discriminating between candidate distributions to describe regional data [44]. However, Hosking & Wallis [16] and Peel et al. [44] pointed out that the L-moment ratio diagrams should not be the sole criterion when choosing the best-fit distribution. They suggested that the criterion should include a goodness-of-fit measure for the identification of acceptable distributions. Accordingly, a distribution was considered acceptable if it satisfies Z^|*DIST*|^≤1.64 (for details, see Hosking & Wallis [16]). If more than one distribution met the criteria, the distribution with minimum Z^|*DIST*|^ was chosen. We show the goodness-of-fit statistics Z^|*DIST*|^ of the five 3-parameter candidate distributions in Table 5. As seen from the table, none of the five distributions is accepted for sub-regions Ⅲ and Ⅳ according to Z^|*DIST*|^. However, the GNO distribution for sub-region Ⅲ and the GLO distribution for sub-region Ⅳ met the principle of the minimum distance between the fitted L-moments and the sample L-moments. If Z^|*DIST*|^> 1.64, the more robust Kappa (four parameters) or Wakeby (five parameters) distributions should be adopted [16]. However, none of them could be well represented on the diagram as each constitutes an area (Kappa) or hyperplane (Wakeby), rather than a line (3-parameter distributions) or a point (2-parameter distributions). Therefore, the Z^|*DIST*|^ criterion is also powerless for Kappa distribution and Wakeby distribution. Here, there is another problem that needs attention. Due to the different indicators or variables selected, various scholars have different results on the climate regionalization of the LP. Based on the SPEI, Liu et al. [8] and Shi et al. [21] used principal component analysis and rotation orthogonal function decomposition methods to divide the LP into southeast, northeast, northwest, and southwest regions, respectively. Another study by Xiao et al. [45] found that the LP may be classified into four distinct climate zones based on its altitude and climate characteristics, namely, semi-humid, semi-arid, arid, and cold-arid. The findings of climate regionalization carried out by Liu et al. [8] and Shi et al. [21] are, for the most part, in agreement with our results, but the results obtained by Xiao et al. [45] differed substantially from ours. In fact, the extent of the sub-humid region is roughly equivalent to the sum of sub-regions Ⅲ and Ⅳ in our study, whereas region Ⅱ roughly includes the arid and semi-arid regions in the study by Xiao et al. [45]. Regardless, differences in climate regionalization will inevitably affect the selection of regional probability distribution, which in turn will influence the results of estimated return periods of annual precipitation in the LP.

**Table 5:** Goodness-of-fit measure Z^|*DIST*|^ for the four homogeneous sub-regions. Bold indicates acceptable distributions.

Sub-region	Distribution				
	GLO	GEV	GNO	PE3	GPA
Ⅰ	10.67	**0.77**	2.26	1.86	-18.44
Ⅱ	13.15	**1.34**	3.34	3.00	-21.31
Ⅲ	8.68	-3.87	-2.34	-3.10	-28.63
Ⅳ	2.46	-5.38	-4.01	-4.21	-20.37

The RFA-LM method may not provide reasonable estimates of the probability of extreme events because L-moments are less susceptible to outliers and fluctuations on the distribution’s tail. Maeda et al. [10] also acknowledged this drawback. They pointed out that assessments of precipitation intensity related to more extended return periods are always associated with higher uncertainty levels. In addition, as mentioned above, the choice of regional probability distribution will also introduce some errors. To some extent, high-resolution gridded data can solve the problem of the uneven distribution of measured sites. However, the gridded precipitation dataset used in this study was also generated by interpolation. Due to the transformation of the data structure, there must be some uncertainties in the results, especially for the area with fewer gauged sites. Further studies should be undertaken to evaluate the uncertainties in areas with lower gauge density using other gridded climate datasets (e.g., reanalysis data and satellite measurements) combined with the L-moment method.

## Conclusions

This paper employed the gridded precipitation dataset obtained from the China Meteorological Data Network to present a spatially explicit characterization of precipitation frequencies over LP based on the L-moment method. The results of this study indicated that the precipitation maps associated with different return periods have a spatial pattern similar to the spatial distribution of MAP. Nevertheless, the amount of annual precipitation increases with the increasing return period. In addition, the frequencies of 80%-of-normal droughts showed a slight difference throughout the area, while those of 60%-of-normal droughts demonstrated substantial differences between the northwestern arid region and the southeastern semi-humid zone. The maps associated with various return periods of precipitation deficits also enable us to identify locations with higher drought frequencies, which can be used to produce drought risk maps together with drought vulnerability maps. Moreover, the maps obtained also imply that different drought thresholds should be used for the different impacts of severe droughts on the southeastern and northwestern parts of the study area. In general, the findings of this research could provide useful insights for drought management, water resource management and decision-making regarding food security policies.

With regard to the selection of the regional probability distribution, a limitation needs to be acknowledged. The GNO distribution for sub-region Ⅲ and the GLO distribution for sub-region Ⅳ did not perfectly meet the Z^|*DIST*|^≤1.64 criterion, which may result in some bias for this study.

## Supporting information

S1 Data Gridded annual precipitation in the Loess Plateau from 1961 to 2017. (ZIP)

S2 Data Loess Plateau boundary. (ZIP)
