# Supplementary figures and images for: Application of L-moment method for regional frequency analysis of meteorological drought across the Loess Plateau, China
Source: PLoS One. 2022 Sep 1;17(9):e0273975. doi: 10.1371/journal.pone.0273975 (PMC9436156; doi:10.1371/journal.pone.0273975)

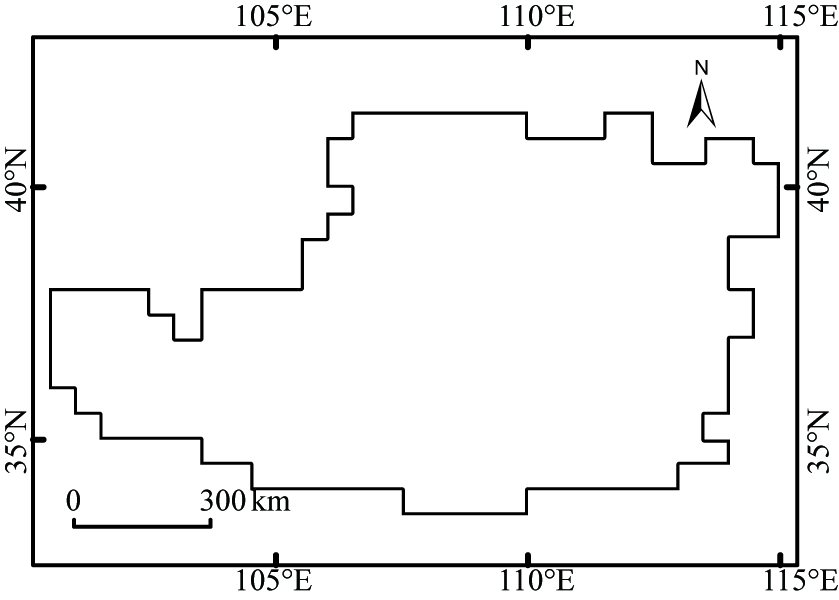

Supplement: S2 Data — (ZIP) [file pone.0273975.s002.zip › LoessPlateauboundary.tif]
